# A NK Cell Odyssey: From Bench to Therapeutics Against Hematological Malignancies

**DOI:** 10.3389/fimmu.2022.803995

**Published:** 2022-04-14

**Authors:** Veronica Ramos-Mejia, Jose Arellano-Galindo, Juan Manuel Mejía-Arangure, Mario Ernesto Cruz-Munoz

**Affiliations:** ^1^ GENYO: Centro Pfizer, Universidad de Granada, Junta de Andalucía de Genómica e Investigación Oncológica, Granada, Spain; ^2^ Unidad de Investigación en Enfermedades Infecciosas, Hospital Infantil de México “Dr. Federico Gomez”, Ciudad de México, Mexico; ^3^ Genómica del Cancer, Instituto Nacional de Medicina Genómica (INMEGEN) & Facultad de Medicina, Universidad Nacional Autónoma de México, Ciudad de México, Mexico; ^4^ Facultad de Medicina, Universidad Autónoma del Estado de Morelos, Cuernavaca, Mexico

**Keywords:** NK cell, cell signaling, cancer, hematological malignancies, immuno-therapy

## Abstract

In 1975 two independent groups noticed the presence of immune cells with a unique ability to recognize and eliminate transformed hematopoietic cells without any prior sensitization or expansion of specific clones. Since then, NK cells have been the axis of thousands of studies that have resulted until June 2021, in more than 70 000 publications indexed in PubMed. As result of this work, which include approaches *in vitro*, *in vivo*, and *in natura*, it has been possible to appreciate the role played by the NK cells, not only as effectors against specific pathogens, but also as regulators of the immune response. Recent advances have revealed previous unidentified attributes of NK cells including the ability to adapt to new conditions under the context of chronic infections, or their ability to develop some memory-like characteristics. In this review, we will discuss significant findings that have rule our understanding of the NK cell biology, the developing of these findings into new concepts in immunology, and how these conceptual platforms are being used in the design of strategies for cancer immunotherapy.

## Introduction

The natural cytotoxicity of NK cells is based on the directed release of the content of lytic granule rich in granzymes, delivered into the cytosol of the target cell through perforin pores assembled at the target cell membrane. This response is mediated and regulated by different types of cell surface receptors. When this cytotoxicity is triggered by NK cell Fc receptors that recognize IgG antibodies bound to a target cell, it is named antibody-dependent cellular cytotoxicity (ADCC). NK cell lytic function is a complex process that involves a series of coordinated events regulated by different receptors and signaling proteins (1, 2). Cell adhesion plays a central role by securing stable contacts between NK cells and target cells and guiding further cellular events. This adhesion step is followed by a stepwise movement of lytic granules to the microtubule-organizing center (MTOC), which then guides lytic granules towards the contact site of the target cell. The highly-organized movement of lytic granules along cytoskeleton elements has been described as granule polarization. Once lytic granules have positioned beneath the plasma membrane, their content is released into a well-defined secretory cleft formed at interface with the target cell, a cellular event defined as degranulation or exocytosis of lytic granules. NK cells can also kill stressed cells through engagement of CD95L and TNF-related apoptosis-induced ligand (TRAIL), which are ligands for the death receptors CD95 and TRAILR respectively (3, 4). Death receptor activation induces the formation of the death-inducing signaling complex that leads to activation of caspase-8 and -10, and ultimately to apoptosis.

In humans, NK cell development initiates with the appearance of the NK-cell precursor (NKP) which arises from a multipotent hematopoietic precursors and early lymphoid precursors (ELP). The NKP give rise to immature NK cells, which in turn are the precursors of more mature NK cells. During maturation, NK cells acquire the expression of various NK cell receptors including those for self MHC class I molecules. The bone marrow represents the main tissue where NK cell development occurs, both in human and mice. By analyzing the phenotype of immature and mature NK cells, different models have proposed the distinction of several stages for developing NK cells (5). Immature NK cells exist in the bone marrow but they have been also found in other tissues including the peri-natal liver in mice and lymph nodes in human, favoring the idea that multiple sites can sustain NK-cell differentiation. An alternative hypothesis sustains that once generated in bone marrow, NKP and immature NK cells access to circulation and populate different peripheral tissues. Once NK cells have gain functional competence, they can be resident cells in various peripheral tissues. However, it is still poorly understood how NK cell maturation is regulated. In mice, mature NK cells can be found in spleen, liver, lung and blood and, to a lesser extent in the bone, marrow, thymus and lymph nodes. In human, mature NK cells represent a substantial fraction of circulating lymphocytes (up to 20%) but they are less frequent in the spleen and bone marrow (5-10%) (6). In contrast, other circulating innate lymphoid cells (ILCs) subsets are found at very low frequencies (less than 0.2%) as they are predominantly found in tissues. In circulating lymphocytes, two major human NK cell subsets are defined by the differential cell-surface expression of CD16 and CD56. CD16^+^CD56^dim^ is the most abundant subset, with high expression of perforin and enhanced cytotoxicity, whereas the CD16^-^CD56^high^ subset is the less abundant and produce greater amounts of IFN-γ and TNF-α (7, 8). The phenotype and function of both NK cell subsets can be modified, as for example, cytokine stimulation downregulates CD16 expression and upregulates CD56 and CCR7 expression. Moreover, cytokines can greatly impact on cytotoxicity and cytokine secretion in CD16^-^CD56^high^ and CD16^+^CD56^dim^ respectively (7). Whereas the phenotype of circulating NK cells is frequently described in terms on CD56 density, NK cell phenotype and function can be shaped through life in response to genetic and environmental factors (8, 9).

NK cells can adapt their behavior to environmental “clues”, such as cytokines present in the milieu or those produced by chronic virus infections. This adaptation may result in enhanced NK functionality and, due to the acquisition of new phenotypic and functional attributes, these NK cells are referred as “memory like” cells. In humans, infections with HCMV are associated with an increased in the percentage of NK cells expressing high levels of the NKG2C receptor (10). In addition, CD16 engagement by anti-HCMV antibodies favors the preferential expansion of adaptive NK cells that upregulated the expression for NKG2C (8, 11, 12). Moreover, other NK cell subsets lacking key B-cell and myeloid-cell signaling proteins, such as FcϵRIγ, SYK, and EAT-2, have also been identified in HCMV-infected individuals (8, 9). Importantly, most of these phenotypic changes are due to epigenetic modifications, providing a mechanism for altered signaling and function in adaptive NK cells compared to conventional NK cells. Whether adaptive NK cells emerge in other scenarios either in health or disease remains to be determined. All these studies clearly indicate that NK cells, as other immune cells, including macrophages and innate lymphoid cells, show high plasticity in response to diverse environmental stimuli such as antigens, acute viral infections, and cytokines (13, 14).

## Specificity For NK Receptors

**Figure 1:**
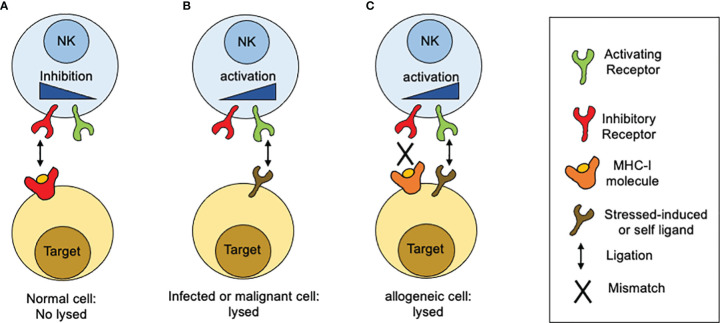
How MHC-I molecules regulate NK cells reactivity. The idea of the missing-self was developed from different observations related with sensing MHC-class I molecules. **(A)** Normal or unstressed cells express MHC-I molecules which are recognized by inhibitory receptors expressed on the NK cell surface. When such receptors are engaged by MHC-I molecules, inhibitory signals are displayed. In the absence of ligation for activating receptors, the inhibitory signals predominate and target cells are not lysed. **(B)** When targets cells NK cells downregulate MHC class I expression, as consequence of viral infections or malignant transformation, inhibitory receptors are no longer engaged. In contrast, target cells start to upregulate the expression of surface molecules that are recognized by activating receptors. As consequence, activation signals are displayed unrestricted and target cells are lysed. **(C)** In the setting of allogeneic recognition, MHC class I molecules are expressed on target cells but are not recognized by NK cells as result of a mismatch. In consequence, activation signals proceed unrestricted and target cells are lysed.

In contrast to T lymphocytes and macrophages, NK cell cytotoxicity does not need prior sensitization or require proliferation of specific clones. When discover, NK cell cytotoxic activity seemed to be independent of antigen receptors and complement receptors. A first clue of NK cell specificity came from the “hybrid resistance” phenomenon, where a F_1_ hybrid mice host rejects a graft derived from either inbred parent but not from an F_1_ hybrid, a rejection that was not predicted to occur according to the laws of transplantation settled at that time (15). This graft rejection was shown to be thymus-independent and mediated by NK cells (16–18). The “hybrid resistance” implies that the absence of at least one major histocompatibility complex (MHC) class I (MHC-I) allele, is sufficient for NK cells from the recipient to eliminate cells “missing” such given MHC-I molecules. Thus, it was proposed that NK cells were able to sense the presence of a whole set of MHC-I molecules (haplotype) in the graft and that their absence would somehow stimulate NK cells. This idea was in line with an earlier suggestion by Snell that rejections of hematopoietic transplants occurs when donor and host differ genetically at the major histocompatibility complex (19). A second hint came from the study on the lifestyle of colonial tunicates, where individual cells are allowed to fuse in order to form colonies. In these invertebrates, the rejection or acceptance between colonies is controlled by a single gene locus with multiple alleles (20). Although the presence of MHC-like molecules is not documented in tunicates, it was possible to stablish an analogy based on the presence of molecules that determine histocompatibility. Despite these clues, there were still some contradictory observations about the relation between MHC-I molecules and NK cell specificity. The most notorious was that the *in vitro* killing of autologous cancer cells by NK cells, which appeared to have no relation to the MHC I molecules expressed on targets cells. Comparing the susceptibility of various cancer cell lines to NK-mediated, allowed to conclude that high levels of MHC class I expression on target cells was sufficient to explain the resistance of targets to be lysed by NK cells. Thus, altering the expression of MHC class I molecules, by inducing cellular stress (neoplastic transformation) or a mismatch for MHC class I recognition, it was possible to tune the susceptibility of target cells to NK cell-mediated lysis. All these observations contributed to build up a model that allowed to explain why NK cells were able to kill “spontaneously” certain target cells (**Figure 1**). Such model was postulated as the “missing-self” hypothesis (21). According to the missing-self hypothesis, NK cells recognize and eliminate cells that have downregulated MHC class I expression, as consequence of viral infections or malignant transformation. At the same time, it was possible to interpretate the fact that metastatic tumor cells could escape from NK cell surveillance by re-expressing MHC class I molecules, lost by the primary tumor (22). All these advances paved the way to the next fundamental breakthrough: the discovery and characterization of inhibitory receptors for self-MHC-I.

## NK Cell-Based Inhibition in the Immune System

The ability of the FcγRIIB receptor to shut down signaling through B cell receptor (BCR) provided the first model to understand how inhibition operates in immune system. The immunoreceptor tyrosine-based activation motif (ITIM) is a 13 amino acid sequence located in the cytoplasmic tail of the FcγRIIB, and is responsible for BCR signaling inhibition (23–25). This sequence resulted to be a specific binding site for the inositol phosphatase SHIP1. This ITIM sequence was reminiscent of a half-ITAM (Immunoreceptor Tyrosine-based Activation Motif), which allows activating signaling through various immune receptors (26). Further studies demonstrated that FcγRIIB was able to inhibit not only BCR-dependent signals but also those emanating from the T cell receptor (TCR) and the high-affinity IgE receptor or FcεRI, suggesting that the inhibitory properties of the FcγRIIB were not limited to any specific cell and could be exerted to any ITAM-containing receptor (25, 27).

Receptors for MCH class I molecules in human NK cells also contained an ITIM sequence (28, 29). Further studies demonstrated that these receptors were able to inhibit NK cell activation upon binding to different MHC class I alleles (30). Unlike ITIMs found in the cytoplasmic tail of FcγRIIB, two YxxL motifs separated by 26 amino acids were present in the cytoplasmic domain of inhibitory receptors for MHC-I molecules (31). A deeper characterization of the two ITIMs in the cytoplasmic tail of the NK cell ITIM-containing receptors (ITIM-Rs) revealed a selective binding motif for SH2 domains of the tyrosine phosphatases, SHP-1 and SHP-2. The selectivity of such inhibitory NK cell receptors for binding SHP-1 and SHP-2 was demonstrated for human and mice. Even thought the recruitment of SH2-containing phosphatases to phosphorylated ITIMs provided a mechanism to explain the inhibitory role of NK cell receptors for MHC-I, this was not sufficient to explain how NK cell ITIMs-R dampen signaling through activating receptors. Two possible mechanisms for phosphatases activity could be envisioned: either tyrosine phosphatases would mediate dephosphorylation of a large number of substrates, which are recruited and positioned through activating receptors, or their phosphatase activity displays a more sophisticated mechanism that targets only key signaling elements that control activation signals. Further studies suggested that inhibitory receptors in NK cells are more suited to block specific signaling events (32). The adaptor protein linker for activation of T cells (LAT) and PLC-γ are SHP-1 substrates, and it has been proposed that NK cell inhibitory receptors focus on regulating the function of adapter LAT rather than directly inhibiting PLC-γ tyrosine phosphorylation (33). Thus, by targeting adapter proteins rather than signaling enzymes, NK cell inhibitory receptors are able to influence multiple stimulatory pathways simultaneously instead of specific signaling pathways. Inhibitory signaling involves the dephosphorylation of Vav1, the only signaling protein associated with the catalytic site of SHP-1 upon recognition of target cells expressing HLA molecules (34). Moreover, the recruitment of Vav to the receptor is independent of actin polymerization, suggesting that inhibitory receptors can dampen NK cell-mediated cytotoxicity through an actin polymerization-independent mechanism (34). Inhibitory receptors can also promote the tyrosine phosphorylation of the adaptor protein Crk and its association with the tyrosine kinase c-Abl, concomitant with its dissociation from c-Cbl and p130 Cas (35, 36). These findings suggested that inhibitory receptors do not only control NK cell activation by dampening tyrosine phosphorylation of signaling proteins but also by promoting tyrosine phosphorylation of downstream signaling elements. How inhibitory receptors promote tyrosine phosphorylation of Crk, and how the association of Crk with c-Abl contribute to inhibit NK cell activation remains to be determined.

**Figure 2:**
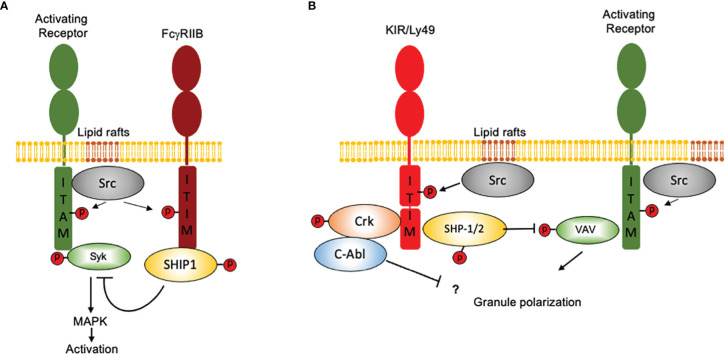
How NK cells contributed to our idea of “inhibition” in the immune system. Inhibition in immune cells is based on the presence of ITIM-containing molecules and in the recruitment of SH2 domain-containing protein phosphatases. Although the presence of an ITIM sequence may represent a basic operation mode for inhibition, there are important differences in how signals are displayed downstream ITIM phosphorylation. For simplicity, only two major systems of inhibition are depicted. **(A)** The FcγRIIB receptor binds a single-SH2 domain-containing phosphatidylinositol 5-phosphate (SHIP1). Activation of SHIP-1 leads to dephosphorylate various substrates downstream ITAM-containing receptors. FcγRIIB coaggregation with activating receptors is mandatory to allow Src family kinases to phosphorylate both, the ITAMs and the ITIMs. **(B)** Inhibitory NK cell receptors bind a two-SH2 domain containing tyrosine phosphatase (SHP1/2). ITIM-containing NK cell receptors are constitutively associated with Src family kinases and as consequence they can signal independently of activating receptors ligation However, coaggregation of an inhibitory NK cell receptor with activating ITAM-containing receptors is necessary to inhibit cell activation. For ITIM-containing NK cell receptors, Vav1 seems to be the only signaling protein associated with the catalytic site of SHP-1 upon recognition of target cells expressing HLA molecules. Moreover, NK cell inhibitory receptors seem to operate no only by promoting tyrosine dephosphorylation, since they are also able to promote the tyrosine phosphorylation of the adapter protein Crk and to promote its association with the tyrosine kinase c-Abl.

In contrast to FcγRIIB, which binds a single-SH2 domain-containing phosphatidylinositol 5-phosphate (SHIP), inhibitory NK cell receptors bind a two-SH2 domain containing tyrosine phosphatase (SHP1/2) (31, 37, 38). Moreover, ITIM-containing NK cell receptors are constitutively associated with Src family kinases and as consequence they can signal independently of activating receptors ligation (39). However, co-aggregation of an inhibitory NK cell receptor with activating ITAM-containing receptors is necessary to inhibit cell activation **(** **Figure 2** **)**. Therefore, NK cells represent a unique model not only to understand the mechanisms of cell regulation by inhibition, but also to appreciate how inhibitory receptors maintain homeostasis and contribute to pathology.

## Inhibitory NK Cell Receptors for MHC-Class I Molecules: Structure and Specificity

In human, MHC-I genes are also designed as human leukocyte antigen (HLA). The cloning of human NK cells showed that NK cells clones displayed a spectrum of different cytotoxic activities towards allogeneic target cells (40). These studies suggested that NK cells were able to recognize different isoforms of HLA class I molecules and the existence of a NK cell repertoire with a given reactivity towards such allogeneic cells (41, 42). The immunization of mice with different NK cell clones allowed the generation of monoclonal antibodies that led to the identification and characterization of NK cell receptors specific for HLA-I molecules. Several studies using such monoclonal antibodies demonstrated that: (i) the expression of NK cell receptors for self HLA-I is maintained unaltered upon cell activation, proliferation or cloning; (ii) the repertoire of NK cell receptors is compound of different members belonging to the same molecular species; and (iii) receptors for HLA-I alleles display different frequencies of expression not only among NK cells from the same individual, but also among different donors (43).

Most of the NK cell receptors for MHC class I molecules, both in human and mice, belong to two major families, which major difference lies in whether the extracellular domain possess a carbohydrate-recognition domain of C-type lectins, or an immunoglobulin-like domains. Gene families that encode for C-type lectins-like receptors are members of the NK complex (NKC) (44), whereas those that encode for immunoglobulin-like receptors belong to the leukocyte receptor complex (LRC) (45). Beyond these differences in structure and genetic organization, both families of receptors have provided two structurally unrelated but functionally complementary systems of inhibitory receptors for MHC-I molecules that drive NK cell development and function.

In humans the LRC includes members of the family of killer cell immunoglobulin-like receptors (KIR), leukocyte Ig-like receptors (LILRs), and leukocyte-associated Ig-like receptors (LAIRs). In humans, the *KIR* gene family contains 15 genes and 2 pseudogenes that are cluster in the chromosome 19q13.4. All members of this family differ for the number of extracellular Ig-like domain and the length of their cytoplasmic tail. In consequence KIR are characterized by the presence of two (KIR2D) or three (KIR3D) Ig-like extracellular domains and by long (KIR2DL and KIR3DL) or short (KIR2DS and KIR3DS) cytoplasmic tails (46). KIRs also contain activating forms that lack ITIMs in their cytoplasmic tails (47, 48). Compared to others NK cell receptor families, KIRs display unique features such as the number of KIR genes present in the genome of any given individual varies within the population (haplotype), the existence of a high polymorphism for each KIR gene, and the stochastic and variegated expression of the KIR repertoire among NK cell subsets. The major consequence of having a variable gene content and allelic polymorphisms for KIR receptors is that unrelated individuals rarely share identical KIR genotypes (49) and that all ethnic populations have their own distribution of KIR-genotypes frequencies (50). Moreover, the number of KIR genes varies between primates, ranging from polymorphic single-copy genes to complex multigene families that result in high levels of haplotypic complexity. In human, there are more than 130 KIR haplotypes that can be divided in at least two distinct groups designated as A and B according to the genes they contain (51). Whereas the former has a common organization of seven genes and two pseudogenes, the latter contains more variable gene content. In general, B haplotype contains more genes that encode for activating KIRs than A haplotype. In contrast to humans, mice and rats encode a single or no KIR gene at the LRC, but they encode for multiple Ly49 genes at the NKC, which provide a similar NK cell receptor system for recognizing missing-self of MHC-I molecules. Despite their differences in protein structure and phylogenies, the KIR and Ly49 gene families arose from gene duplication and diversified by gene conversion, both families are highly polymorphic, expressed in NK cells and some T cell subsets, and their expression in NK cells is influenced by the host MHC class I haplotype.

In humans, KIR genes are expressed in variable combination by NK cells and some subsets of effector/memory αβ T cells, γδ T cells, but absent in thymocytes or naïve T cells (52, 53). Members of the KIR family include receptors for of HLA-A, HLA-B, and HLA-C proteins, which are also the products of highly polymorphic genes that are cluster on chromosome 6. However, the expression of KIRs does not depend on the expression of their HLA class I ligands and as a result, a given individual may express KIRs with no specificity for self-HLA alleles (51). The inhibitory receptors KIR2DL1, KIR2DL2, and KIR2DL3 recognize a polymorphism in HLA-C molecules which are distinguished by the presence of Ans77 and Lys80 or Ser77 and Asn80 on the α1 domain of the HLA-A heavy chain. In addition, the inhibitory receptors KIR3DL1 and KIR3DL2 recognize HLA-B and HLA-A molecules respectively. Variation at position 80 of HLA-C defines two groups of KIR ligands, the MHC-C1 allotypes (Asn80), and MHC-C2 allotypes (Lys80). The KIR2DL2 and KIR2DL3 receptors are specific for group C1, whereas KIR2DL1 is specific for group C2. The consequence of this natural variation at position 80 of HLA-C is an alteration in the specificity and strength of the HLA-C binding by KIR members. The interaction of C2 with KIR2DL1 is stronger and more specific than that of C1 with KIR2DL2 or KIR2DL3. However, both C allotypes are well represented in all human populations suggesting that both allotypes may have complementary functions providing variation in the HLA-C-mediated inhibition. As for HLA-C, HLA-B also presents a dimorphism in the C-terminal region of the α1-helix that determines the specificity for binding of inhibitory KIRs. As a result, two epitopes defined as Bw4 and Bw6 have been identified (54), but only Bw4 function as a KIR ligand which is recognized by KIR3DL1 (55, 56). Both epitopes are also present in all human populations suggesting complementary functions.

Besides members of the LCR family, the NKC also encodes for receptors that bind MHC class I molecules. These lectin-type receptors are type II transmembrane proteins and the inhibitory and activating CD94/NKG2 receptors. In contrast to the evolutionary dynamic KIR system, the CD94:NKG2 is an older and more conserved system. CD94 associates with various NKG2 receptors such as NKG2A, NKG2C and NKG2E. In contrast, NKG2D is a homodimer. In contrast to human, which only encodes a single Ly49L pseudogene, the NKC of mouse includes the Ly49 family of lectin-type NKRs (44, 57). The expansion of Ly49 genes is only observed in rodents and horse. Of the three heterodimers, only NKG2A contains and ITIM motif and as consequence, is able to trigger inhibitory signals that tune NK cell activation (58). In contrast, NKG2C and NKG2E, by mean of a charged residue in their cytoplasmic tail, are able to associate with DAP-12 and to transmit activating signals (59, 60). The binding partner for NKG2A and NKG2C is the non-classical MHC class I molecule HLA-E (61, 62). HLA-E bind peptides derived from the leader peptide of HLA class I sequences (61, 63), which contain polymorphisms that either enhance or diminish peptide binding to HLA-E. In addition, HLA-E can bind heat-shock-protein derived peptides (64), viral peptides (65), and other ligands that also influence NK cell sensitivity (66). Because a proper folding and cell surface expression of HLA-E is dependent on such peptide binding, the surface abundance of HLA-E detected by CD94/NKG2A correlates with the amounts of HLA-A, HLA-B, and HLA-C being produced by any given cell. Therefore, polymorphism of HLA class I has important implications in NK cell development and function. A particular dimorphism in the leader peptides of HLA-B modulates its binding to HLA-E. Binding of this peptide to HLA-E is stronger when a methionine is at position -21 of the HLA-B leader sequence that when a threonine is at the same position (67). Based on this HLA-B dimorphism, humans are divided in three groups (M/M, M/T, and T/T). Those groups containing the M/M and M/T haplotypes have CD94-NKG2A+ NK cells that also express a repertoire of cell surface receptors more diverse and NK cell effector function are more potent than those NK cells form T/T haplotype (68). Thus, a simple dimorphism in HLA-B has had in important influence in NK cell function and diversity (68). Despite KIR and CD94/NKG2A evolved independently, they complement each other to direct NK cell development and function during immune response.

## Activating NK Cell Receptors

Receptors can favor NK cell activation through any of the following mechanisms: adhesion, granule polarization, and degranulation. Members of the natural cytotoxic receptor (NCR) family are among these receptors, including Nkp30 (69), Nkp44 (70, 71), (only in humans), and Nkp46 (72, 73). Members of this family are associated with ITAM-containing adapter transmembrane proteins such as CD3-ζ, FcϵRIγ, and DAP-12. Many ligands have been identified for these receptors including viral and bacterial proteins, as well as cell surface endogenous molecules, although the physiologic relevance of these interactions remains uncertain. Other important activating receptors include NKG2D (74) and DNAX accessory molecule (DNAM)-1 (75). NKG2D is a C-type-lectin receptor, which recognizes several MHC class I-related ligands that are induced in cells undergoing viral infection or malignant transformation. DNAM is an activating receptor that binds to the poliovirus receptor CD155 and to the nectin adhesion molecule CD112, both of which are up-regulated in cancer cells. In consequence, DNAM has an essential role in preventing spontaneous tumor formation and in controlling tumor growth (76, 77) 4. DNAM promotes NK cell activation *via* an immunoreceptor tyrosine tail (ITT)-like motif that couplesDNAM-1 to Grb2 and to downstream effectors such as Vav-1 (78). Even though all these receptors contribute with positive signaling, none of them activate a full-cytotoxic function by itself.

Therefore, all these receptors can interplay and cooperate to overcome a critical threshold that counterbalances the effect of inhibitory receptors. In contrast, CD16, the low-affinity receptor for IgG (FcγRIII) and the mediator of antibody-dependent cellular cytotoxicity (ADCC), is the only receptor that seems to fulfill the necessary requirements to promote strong effector functions without requiring synergy through costimulatory receptors, although the molecular mechanism underlying this function remains unknown (79, 80) 47,48. CD16 is found to be associated with FcϵRIγ chains or CD3ζ chains either as homodimers or heterodimers, each one containing ITAM motifs.

Beside receptors with a well-defined function of activating or inhibiting, NK cells also express receptors with the dual capability to activate or inhibit NK cell-mediated functions. The prototypic receptors with this “dual” function are those grouped in the SLAM family. This family belongs to the Ig superfamily and is composed by 6 members, named SLAM, CD244, Ly9, NTBA, CD84, andCD319 (81). Apart from CD244 (2B4), which recognizesCD48 (other member of the SLAM family) as a ligand, all members of this family are self-ligands and participate in the context of heterotypic or homotypic cell interactions. The expression of these receptors is restricted to the hematopoietic compartment. All members of the SLAM family harbor at least one tyrosine-based motif named immunoreceptor tyrosine-based switch motif (ITSM). This motif plays an essential role in determining the signals that are delivered downstream of the cytoplasmic tail of SLAM receptors. The tyrosine residue present in the ITSM motif is a bona fide site for interaction with members of the SAP family (82). In addition to the tyrosine residue of the ITSM motif, the cytoplasmic tail of SLAM receptors also harbors tyrosine residues that are potential sites for phosphorylation by Src family kinases. As a consequence, these tyrosine residues become sites for recruitment of SH2 domains-containing phosphatases. When these receptors are engaged, the SLAM receptors deliver signals that favor NK cell activation, in part by regulating inside-out signaling through LFA-1 or by enhancing granule polarization (83). In contrast, when SLAM receptors are engaged in NK cells lacking expression of SAP adapters, the signals emanating downstream lead to the recruitment and activation of SHIP1, which in turns favors Vav-1 dephosphorylation, a key element required to mediate NK cell activation. Therefore, SAP adapters are critical elements that regulate NK-mediated functions in part by uncoupling SLAM receptors from recruiting phosphatases such as SHP-1 and SHIP1.

## NK Cell Reactivity and Self-Tolerance

The missing-self hypothesis explained the close relation between inhibitory receptors for MHC-I molecules and NK cell reactivity and further studies allowed the following conclusions: (i) NK cells simultaneously express multiple inhibitory receptors in a random manner and some of these are specific for self-MHC class I, (ii) a unique NK cell repertoire is influenced by the extension of the number of MHC class I alleles in the host, and (iii) not all NK cells display the same reactivity towards allogeneic target cells (84, 85). Nevertheless, various studies also demonstrated the existence of NK cells lacking expression of inhibitory receptors with specificity for self-MHC class I (86), and according to the missing self-hypothesis, NK cells with no expression of inhibitory receptors for self-MHC class I, loss tolerance in an MHC class I-deficient host. However, observations made in humans who has loss the expression of MHC I molecules due to genetic errors, indicated that NK cells do not spontaneously overreact against the self (87). Same observations were obtained from studies in mice deficient for B2-microglobulin (β_2_m) or transporters associated with antigen presentation (TAPs) (88–92). In contrast to expected, NK cells from MHC class I-deficient mice displayed a poor cytotoxicity activity towards MHC class I-deficient targets, despite they were normal in numbers, tissue distribution and expression of activating receptors. Moreover, NK cells from a host lacking MHC-class I expression, were unable to reject MHC class I-deficient bone marrow grafts compared to NK cells from MHC class I-sufficient mice. More detailed studies assessing NK cell responses on a per cell basis, found that NK cells expressing an inhibitory receptor for self-MHC class I displayed efficient effector functions in response to stimulation through activating receptors, but NK cells lacking such inhibitory receptors failed to mount effector responses (93). Moreover, the presence of MHC-I ligands increased the frequency of human NK cells expressing KIR family members, suggesting that frequencies of NK cell subsets expressing inhibitory receptors for MHC-I molecules is shaped by the self-MHC-I environment (94). Therefore, the capacity of a given individual NK cell to become functionally competent was correlated with the expression of an inhibitory receptor with specificity for self-MHC class I. This idea was further supported by studies in transgenic mice for a single-chain trimer specific for the inhibitory receptor Ly49, in which only NK cell expressing Ly49 inhibitory receptor showed a normal production of IFN-γ as compared to NK cells lacking the expression of such receptor. The explanation for this phenomenon was forged under different names including NK cell “education”, “licensing”, “arming”, “disarming”, among others. The basic idea is that mammals have evolved a mechanism by which NK cells, are positively selected during development for the expression of inhibitory receptors that recognize self-class I alleles. If such recognition takes place, then NK cells are allowed to undergo further maturation to become functionally competent. In contrast, when NK cells fail to recognize such self-class I molecules, further differentiation is arrested. This model allows explaining how NK cells become tolerant to self-MHC class I molecules while ensuring reactivity to allogeneic-class I molecules.

**Figure 3:**
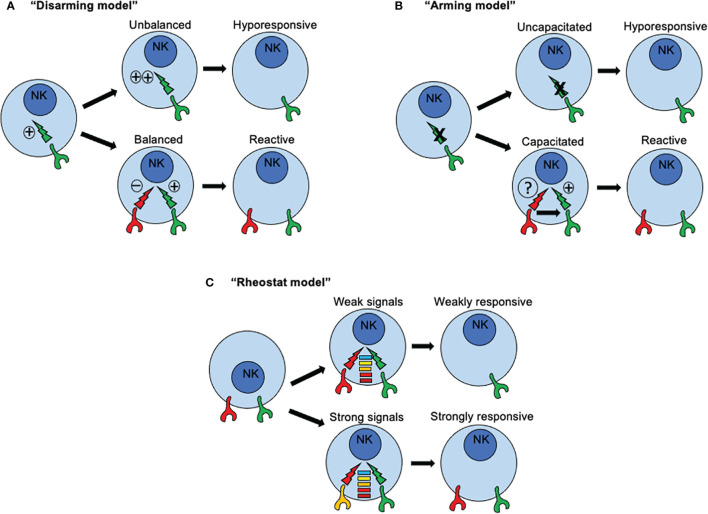
How recognition of MHC class I molecules drive NK cell reactivity and self-tolerance. Three major models have been proposed to explain how NK cells become functionally competent. **(A)** The first model is based on a “disarming” mechanism and propose that activating signals in immature NK cells are by default in “ON” mode. If such NK cells acquire inhibitory receptors, then interactions with MHC class I molecules prevent such default “ON” mode, NK cell tolerance is set-up and NK cells mature normally. However, if such NK cells fail to express inhibitory receptors for self-MHC-I, then prolonged or unopposed activation signals would lead eventually to a state of NK cell hypo-responsiveness. **(B)** The second model, based on a “licensing” or “arming” mechanism, sustains that activating signals are by default in “OFF” mode. When NK cells acquire the expression of inhibitory receptors for self-MHC-I, then the signaling through such inhibitory receptors capacitate activating receptors to become functionally competent. If NK cells fail to express inhibitory receptors, then the activating receptors would remain functionally incompetent. **(C)** A third model known as the “rheostat” model has been proposed to explain how NK cell responsiveness in regulated during NK cell development According to this model, NK signals are not displayed as a binary system (“on” or “off”), but as an analogue system. The latter means that NK cell responsiveness can be “tune up” or “tune off” in a quantitative rather than in a binary manner depending on the inhibitory signal triggered by any particular inhibitory receptor for self-MHC class I ligand.

Three major models have been proposed to explain how NK cells become functionally competent. The first model is based on a “disarming” mechanism and propose that activating signals in immature NK cells are by default in “ON” mode. If such NK cells acquire inhibitory receptors, then interactions with MHC class I molecules prevent such default “ON” mode, NK cell tolerance is set-up and NK cells mature normally (**Figure 3A**). Under this scenario, functional activation signals ensure proper NK cell responses towards cells that have loss or downregulated MHC-I expression. However, if such NK cells fail to express inhibitory receptors for self-MHC-I, then prolonged or unopposed activation signals would lead eventually to a state of NK cell hypo-responsiveness. Conversely, the second model, based on a “licensing” or “arming” mechanism, sustains that activating signals are by default in “OFF” mode. When NK cells acquire the expression of inhibitory receptors for self-MHC-I, then the signaling through such inhibitory receptors capacitate activating receptors to become functionally competent (**Figure 3B**). Once stablished, same inhibitory receptors mediating capacitance for activating receptors would restrain activating signals, avoiding self-reactivity. If NK cells fail to express inhibitory receptors, then the activating receptors would remain functionally incompetent.

An essential difference between these two models is the nature of the signals that are triggered by inhibitory receptors for self-MHC-I. In immature NK cells, signaling through inhibitory receptors is dependent on ITIMs present in their cytoplasmic domains. When NK cells express an inhibitory receptor with a mutated ITIM, signals through activating receptors are dysfunctional rendering NK cells functionally incompetent. This dependence of acquiring NK cell reactivity for an ITIM domain is consistent with the two models. However, a qualitative difference for signals through inhibitory receptors is assumed. The default in “ON” mode implies a conventional inhibitory role, whereas the default in “OFF” mode connotes a new instructive role for inhibitory receptors. However, only the default in “ON” model assumes that a continuous and unrestrained signaling through activating receptors would induce an anergy-like responsiveness. A similar scenario was described in NK cells exposed to NKG2D ligand-expressing tumor cells *in vitro* (95), or enforced expression of NKG2D ligand *in vivo* (96), where continuous engagement by such ligands render NKG2D signaling dysfunctional. Same outcomes were found in mice expressing a viral protein from MCMV, where continuous engagement of a self-specific activating receptor during development induces NK cell tolerance (97, 98). Interestingly, the continuous NKG2D engagement not only affected NKG2D signaling but also those triggered by other activating receptors. For example, the signals through NK1.1 and CD16 also resulted impaired due to sustained NKG2D signaling. These studies suggest that a cross-tolerogenic effect can be induced in NK cells (95). Further studies have observed that a single MHC class I allele may impact on different subsets of NK cells according to the repertoire of inhibitory receptors, and that a given subset of NK cells may can be influenced by several different MHC class I alleles. All these observations point to that idea that NK cell education is not an all-or-none phenomenon but this it can be described in quantitative terms (99, 100). As consequence, a third model known as the “rheostat” model has been proposed to explain how NK cell responsiveness in regulated during NK cell development (101). According to this model, NK signals are not displayed as a binary system (“on” or “off”), but as an analogue system (**Figure 3C**). The latter means that NK cell responsiveness can be “tune up” or “tune off” in a quantitative rather than in a binary manner depending on the inhibitory signal triggered by any particular inhibitory receptor for self-MHC class I ligand. This model does not exclude the previous mechanisms proposed.

Although we have learned that self-MHC-I molecules play a central role shaping the NK cell repertoire and regulating reactivity and tolerance, several issues remain to be addressed. For example, are NK cells educated only at certain time window during development? Or mature NK cells can be “reeducated”? A clue for this question may come from a recent study where the SLAMF6 receptor was found to be critical to control NK cell responsiveness. NK cells lacking the adapter protein SAP displayed enhanced responsiveness towards nonhematopoietic cells, this effect was also observed in response to the engagement of various activating receptors including DNAM, NKp46 and CD16, a phenomenon also observed during NK cell education mediated by KIRs and Ly49. This effect was no longer observed in the absence of SLAMF6, indicating that this receptor was largely responsible to mediate these effects. Interestingly, the loss of SLAMF6 in the NK cell line YT-S also resulted in a diminished responsiveness towards nonhematopoietic cells. Therefore, this study suggest that education of NK cells may not be restricted to only during NK cell maturation but also may occur in mature NK cells, and that NK responsiveness may not be equally regulated for hematopoietic and nonhematopoietic target cells.

## NK Cell-Based Therapy in Hematological Malignancies

Since their discovery in the 1970s, NK cells have been highlighted by their unique ability to recognize and eliminate cells undergoing neoplastic transformation. NK cells can not only directly kill tumor cells, but also can enhance antibody and T cell responses, therefore NK cell- immunotherapy is emerging as a promising therapeutic approach in cancer. Below we will discuss the major advances in NK cell-based immunotherapy against hematological malignancies.

Early clinical evidence in hematopoietic stem cell transplants (HSCTs) suggested that NK cells in the donor graft recognize and eliminate residual malignant cells (102). These findings encouraged to investigate the NK cells as effectors in immunotherapies against hematological malignancies. However, the clinical use of NK cells faces big challenges such as to obtain sufficient cells for adoptive transfer, the persistence of transferred cells and the low activity of naïve NK cells. In initial studies, NK cells for autologous therapy were expanded and activated *ex vivo* using interleukin-2 (IL-2), and even though the infusion of activated NK cells proved to be safe, it did not improve patient outcome (103). In a different approach, haplotype-mismatched HSCTs showed that NK cells exert potent antileukemic effects and did not cause graft-versus-host disease (GvHD), preventing leukemia relapse in acute myeloid leukemia (AML) patients (104, 105). This graft versus leukemia (GvL) effect is associated with a mismatch between host HLA groups and donor NK cell KIR ligands, in which the lack of “self” MHC class-I molecules on leukemia cells is crucial to unrestraint NK cell cytotoxicity (104–106). In addition, NK cells can suppress GvH disease by lysing alloreactive T cells and antigen-presenting cells (APCs), or either by inhibiting T cell proliferation (107–109).

In adoptive NK cell therapy, to avoid side effects such as GvHD or B-cell lymphoproliferative disease, is recommended to purify NK cells with minimal contamination by B or T cells (110, 111). A pioneering study of allogeneic NK cell transfer showed that haploidentical NK cells purified from related donors can expand *in vivo* after subcutaneous IL-2 administration and lymphodepleting chemotherapy. Although 30% of poor prognosis AML patients reached complete remission, the high intensive immune suppressive regimens and the high doses of IL-2 resulted in significant toxicities (112). In a posterior study, milder conditioning regimen and purified NK cells without *in vitro* exposure to cytokines allowed the engraftment of haploidentical NK cells and reduced the risk of relapse in pediatric patients with AML (113).

Cytokines such as IL-2, IL-15, IL 18 and IL-21 promote the proliferation, activation and cytotoxicity of NK cells against leukemia cells (112, 114–118). Feeder cells also support the expansion and activation of NK cells, including irradiated PBMCs (116, 119), EBV-LCL Epstein–Barr virus-transformed lymphoblastoid cell lines (EBV-LCLs) (120, 121) or gene-modified K562 cells expressing membrane-bound (mb)IL-15 or mbIL-21 (86, 122, 123). IL-15 has a preponderant role in NK cell function, differentiation, proliferation and survival (124, 125). Preclinical studies showed that IL-15 was able to expand and activate T cells and NK cells *in vivo* (126). IL-15 immunomodulatory properties are being explored alone or in combination with other agents to potentiate anti-tumor responses. IL-15 is frequently found associated to the receptor IL-15Rα on dendritic cells (127). It has been shown that soluble IL-15/recombinant IL-15Rα complexes elicit a rapid and strong expansion and activation of CD8+ T cells and NK cells, improving immune activation *in vivo* (128). The safety and efficacy of superagonist IL-15-IL-15Rα (ALT-803) was investigated in a clinical trial in hematological malignancies relapsed after HSCT. This study demonstrated that ALT-803 selectively expanded and activated CD8+ T cells and NK cells, producing favorable responses in almost 20% of the patients (including 1 complete remission) (129). Currently, some clinical trials in hematological malignancies are investigating safety and efficacy of ALT-803 in combination with NK cell adoptive therapy or antibodies that elicit antibody-dependent cell cytotoxicity (ADCC) (**Table 1**) (clinicaltrials.gov: NCT02384954, NCT01898793 and NCT02782546). In a different study, the recombinant human IL-15 (rhIL-15) receptor agonist NKTR-255 was designed to obtain a sustainable activation of the IL-15 pathway. A phase I study of NKTR-255 as monotherapy and in combination with antibodies in relapsed/refractory hematologic malignancies is ongoing (clinicaltrials.gov: NCT04136756) (130).

**Table 1:** CAR-NK-cell therapy for hematological malignacies registered in clinicaltrials.gov.

Clinical trial identifier	Phase	Disease	Target	NK-cell source	Location
NCT02892695	I/II	Relapsed/Refractory Leukemia and Lymphoma	CD19	NK-92 cell line	China
NCT03056339	I/II	Relapsed/Refractory B-Lymphoid Malignancies	CD19	CB-NK cells	USA
NCT04555811	I	Diffuse large or high-grade B cell lymphoma.	CD19	hiPSC-derived NK cells (FT596)	USA
NCT04245722	I	Relapsed/Refractory B-cell Lymphoma and CLL	CD19	hiPSC-derived NK cells (FT596)	USA
NCT04887012	I	Refractory/Relapsed B-cell NHL	CD19	HLA haploidentical-NK cells	China
NCT04796675	I	Relapsed/Refractory B Lymphoid Malignancies	CD19	CB-NK cells	China
NCT00995137	I	Relapsed/Refractory B-lineage ALL	CD19	HLA haploidentical-NK cells	USA
NCT03690310	Early Phase I	Relapsed/Refractory B Cell Lymphoma	CD19	N/A	China
NCT04639739	Early Phase I	Refractory/Relapsed B-cell NHL	CD19	N/A	China
NCT03824964	Early Phase I	Relapsed/Refractory B Cell Lymphoma	CD19/CD22	N/A	China
NCT03692767	Early Phase I	Relapsed/Refractory B Cell Lymphoma	CD22	N/A	China
NCT02944162	I/II	Relapsed/Refractory AML	CD33	NK-92 cell line	China
NCT04623944	I	Relapsed/Refractory AML or MDS	NKG2D	N/A	USA
NCT03940833	I/II	Relapse/Refractory MM	BCMA	NK-92 cell line	China
NCT02742727	I/II	Relapsed/Refractory Leukemia and Lymphoma	CD7	NK-92 cell line	China

The first in-human trial with activated NK cells using the K562-membrane-bound IL-15 (K562-mb15-41BBL) feeder cell line, derived from HLA-matched donors and infused into patients with high-risk solid tumors, resulted in an unexpected high incidence of acute GvHD, even after T-cell depletion, suggesting that allogenic IL-15/4-1BBL activated NK cells can contribute to GvHD (131). In contrast, adoptive transfer of donor-derived NK cells activated and expanded (NKAE) using the K562-mbIL21 line in patients with hematological malignancies, did not increase the risk of GvHD (132). Furthermore, autologous transfer of NKAE cells using the K562-mIb15-41BBL cell line was well tolerated in patients with a bad prognosis multiple myeloma (MM) and showed clinical efficacy in two of five subjects (133). Currently, some clinical trials in pediatric acute leukemia (clinicaltrials.gov: NCT01944982, NCT02074657 and NCT02763475) are addressing the clinical efficacy of haploidentical NKAE cells in combination with chemotherapy to eliminate chemotherapy-resistant leukemic cells (118, 134). Human NK cells have the ability to retain an intrinsic memory of prior activation after stimulation with cytokines, exhibiting enhanced IFN-γ and TNF production and cytotoxicity upon reactivation (135, 136). In a phase I study of adoptive transfer in poor-prognosis AML patients, memory-like NK (ML NK) cells were generated by pre-activation of haploidentical NK cells with IL-12, IL-15, and IL-18. ML NK cells efficiently expanded *in vivo* and displayed enhanced cytotoxicity against leukemia, and favorable outcomes were observed in five of nine patients, including four complete remissions (117). Preliminary results of other phase I study in patients with relapsed myeloid malignancies after haploidentical donor transplant (**Table 1**) (clinicaltrials.gov: NCT040247761), showed that ML NK cells expanded massively and persisted for several months after infusion (/doi.org/10.1182/blood-2020-133933).

### Sources of NK Cells for Therapy

Early studies in adoptive cell therapy used NK cells derived from peripheral blood (PB) since they are easy to obtain and have a mature phenotype. However, NK cells represent a small and heterogeneous fraction of PB cells with a reduced proliferative capacity, and it is difficult to implement large-scale expansion methods for clinical uses (112, 137, 138). In addition, autologous PB-NK cells have not given the expected results in the clinic since the NK cells from cancer patients are often functionally impaired (103, 137). Therefore, alternative allogenic sources of NK cells are being explored for cancer immunotherapy.

Higher percentage of NK cells can be isolated from Umbilical Cord Blood (UCB), which are easy to collect and cryopreserve, offering an attractive source of NK cells for therapy (139). In one study NK cells derived from cord blood (CB) were expanded and activated *ex vivo* using K562 cells expressing IL-2, and transferred into multiple myeloma patient in combination with chemotherapy and autologous HSCT. This study resulted in 10 of 12 patients achieving a good partial response and revealed that CB-NK cells maintain an active phenotype *in vivo* but did not persist for long-term after infusion (130). Another clinical trial used CD34+ progenitor cells isolated from partly HLA-matched UCB units to generate NK cells. The hematopoietic stem and progenitor NK cells (HSPC-NK) generated *ex vivo* were safely infused in AML patients after immunosuppressive chemotherapy and exerted a transient antileukemic effect. This study demonstrated that HSPC-NK cells engraft and undergo *in vivo* maturation (140).

The NK-92 cell line, derived from a lymphoma patient, is a homogeneous source of NK cells that can be easily genetically manipulated, cryopreserved and expanded with a high purity and function (141). Nevertheless, the NK-92 cell line needs to be irradiated before infusion due to its tumorigenic potential, which limits the *in vivo* survival and expansion of these cells, and very high doses of NK-92 cells are required for allogenic administration (142). A phase I trial for refractory hematological malignancies relapsing after autologous HSCT, demonstrated that irradiated NK-92 cells infusions are safe even at high doses (142). Two more clinical trials are addressing the safety and efficacy of NK-92 cells in hematological malignancies (**Table 1**) (clinicaltrials. gov: NCT00900809 and NCT00990717).

NK cells also can be derived from human pluripotent stem cells (hPSCs) (143). Human PSC-derived NK cells (hPSC-NK) have a potent anti-tumour activity and proliferative capacity, and are easy to genetically modify (143). One major advantage of hPSC-NK cells and NK-92 cells is the possibility to obtain enough NK cells to allow multiple infusions into patients, in a cost effective and standardized manner. However, hPSC-NK cells have the potential for malignant transformation and safety should be careful addressed in clinical trials. Since NK cells have a lifespan of about 2 weeks (144), stimulation with IL-15 has been used to improve the persistence of NK cells after infusion *in vivo* (129). The Cytokine-inducible SH2-containing protein (CIS), encoded by the CISH gene, is a negative regulator of IL-15 signaling in NK cells (145). To improve survival and anti-tumor activity of NK cells derived from human induced pluripotent stem cells (iPSC), the CISH gene was deleted in iPS cells using the CRISPR/Cas9 technology. CISH−/− iPSC-NK cells resulted in increased IL-15 signaling, increased expansion and enhanced cytotoxicity. In an AML xenograft model, CISH−/− iPSC-NK cells had an improved *in vivo* persistence and superior anti-tumor activity in comparison with WT iPSC-NK cells and (146).

However, iNK cells are characterized by a lower CD16 expression compared to PB-NK cells (147). The Fc receptor CD16a is cleaved by the ADAM17 metalloprotease upon stimulation (148). To stabilize the expression of CD16a, iPSCs were genetically engineered to express a high-affinity variant of CD16a with a mutation that confers resistance to ADAM17-mediated cleavage (149) (hnCD16). NK cells derived from hnCD16-iPSC (hnCD16-iNK) were functionally mature, maintained high levels of stable CD16a expression and exhibited enhanced ADCC against multiple cancer cell lines *in vitro*. Pre-clinical xenograft studies with hnCD16-iNK cells and the anti-CD20 antibody led to improved regression of B-cell lymphoma tumors compared with PB-NK cells or unmodified iNK cells (149). A phase I study of hnCD16-iNK cells (FT516) as monotherapy in AML and in combination with an anti-CD20 antibody in B-cell lymphoma is undergoing (clinicaltrials. gov: NCT04023071). Preliminary results in patients with relapsed/refractory B-cell lymphoma showed that two of four patients achieved a complete response and another patient achieved a partial response, with no associated toxicities (Fate-Therapeutics-Reports-Positive-Interim-Data-from-its-Phase-1-Study-of-FT516-in-Combination-with-Rituximab-for-B-cell-Lymphoma.html. Accessed 12 August 2021).

### Monoclonal Antibodies Enhance NK Cell-Mediated ADCC in Hematological Cancers

NK cells are the main lymphocyte subset responsible for the ADCC. In cancer immunotherapy, antibody-dependent tumor killing through NK cells relies in the development of monoclonal antibodies (mAB) directed against highly expressed antigens in tumor cells. The US Food and Drug Administration (FDA) and the European Medicines Agency (EMA) have approved several monoclonal antibodies to mediate ADCC in hematological cancers, used as single agent or in combined regimens. Rituximab was the first mAb approved by FDA for the treatment of Non-Hodgkin lymphoma (NHL) and chronic lymphocytic leukemia (CLL) (150). Rituximab is a chimeric mouse/human anti-CD20 mAb, which mainly acts by inducing ADCC of NK cells and complement-dependent cytotoxicity (CDC) (150). Since approximately half of all patients become refractory to rituximab treatment, new anti-CD20 mAbs with an increased ADCC have been developed, such as Ocrelizumab (151), Obinutuzumab (152) and Ublituximab (153). Alemtuzumab is another mAB approved for the treatment of patients with relapsed/refractory CLL (154). Alemtuzumab targets CD52 and its mechanisms of action mainly rely on NK-mediated ADCC (155). Daratumumab is a human mAB that targets CD38, approved as monotherapy and in combination with standards of care for MM. Preclinical studies have shown that daratumumab induces cell death through mechanisms such as ADCC and CDC (156). Elotuzumab is a humanized mAB that targets the glycoprotein SLAM Family Member 7 (SLAMF7), also known as CS1, approved for the treatment of relapsed and/or refractory MM in combination with other agents. Elotuzumab induces NK cell cytotoxicity against myeloma cells directly thought ADCC or indirectly by facilitating SLAMF7-SLAMF7 interactions between NK cells and myeloma cells to enhance natural cytotoxicity (157).

Furthermore, antibodies directed to immune checkpoint receptors, capable to regulate NK cells response, are being tested in cancer therapy. Lirilumab is a mAB that binds to KIR2DL1 and enhance NK antitumor effect by increasing NK-cell-mediated killing of HLA-C expressing tumor cells (158). Phase 1 clinical trials of lirilumab in hematological malignancies have demonstrated that prolonged KIR blockade is safe and well tolerated by patients (158, 159), and additional studies as single agent or in association with other immunomodulatory drugs are underway (**Table 1**) (clinicaltrials.gov: NCT02252263, NCT01687387). Monalizumab is an anti NKG2A-mAb that block NKG2A-HLA-E binding and restore the function of NK cells by blocking the inhibitory signals given by tumor cells expressing HLA-E (160). Preclinical studies in CLL demonstrated that blockade of NKG2A though monalizumab restores cytotoxicity of NK-cells against HLA-E+ CLL cells (161). An ongoing phase I trial is investigating the safety of monalizumab in hematologic malignancies after HLA matched allogenic HSTC (clinicaltrials.gov: NCT02921685).

### Bispecific and Trispecific Killer Cell Engagers

New NK cell-based therapeutic approaches involve the use of bispecific and trispecific killer engagers, BiKEs and TriKEs respectively, which target CD16 and tumor antigens to induce the formation of immune synapses between NK cells and tumor cells, triggering cytotoxic responses (162). The group of Dr. Jeffrey Miller has developed several BiKEs and TriKEs as potential agents for leukemias and lymphomas immunotherapy. They have demonstrated that the CD16/CD19 BiKE and the CD16/CD19/CD22 TriKE enhanced NK cell cytotoxicity against B-cell tumor cell lines and primary leukemia cells (163). Also a BiKEs that targets CD16 along with CD33 (CD16/CD33) facilitated effective NK cell elimination of primary CD33+ AML and CD33+ MDS patient cells (164, 165). However, they found a direct correlation between NK cell activation and the downregulation of CD16 expression after exposure to the CD16/33 BiKE (164). The same group designed the CD16/IL-15/CD33 TriKE by integrating IL-15 into the CD16/CD33 BiKE, to deliver a proliferation/survival signal to NK cells and enhance the *in vivo* persistence. The CD16/IL-15/CD33 TriKE not only was able to enhance NK function against CD33+ cell lines and AML blasts, but also to expand and sustain human NK cells *in vivo*, reducing tumor burden in a xenograft model (166). This TriKE (GTB-3550) is currently in Phase I/II clinical trial for high-risk hematological malignancies (clinicaltrials.gov: NCT03214666).

### Chimeric Antigen Receptor-Engineered NK Cells

The chimeric antigen receptor (CAR) -T cell therapy has achieved great success treating patients with hematological malignancies (167). The approval of CAR-T cell therapy in the United States, Europe, Canada, Australia and Japan represent a major milestone of genetically modified cell immunotherapy (168). The FDA in the US and the EMA in Europe had already approved three CAR-T products for B-cell malignancies that target the CD-19 antigen: (1) Tisagenlecleucel (KYMRIAH), Novartis Pharmaceuticals Corporation, for relapsed B-cell Acute Lymphoblastic Leukemia (ALL) and relapsed or refractory large B cell lymphoma, (2) Axicabtagene Ciloleucel (YESCARTA), Kite Pharma Inc., for relapsed or refractory large B cell lymphoma, and brexucabtagene autoleucel (TECARTUS), Kite Pharma Inc., for the treatment of relapsed or refractory mantle cell lymphoma.

The CAR is an engineered protein based on the T cell receptor (TCR) that is composed of an extracellular antigen binding domain, followed by a transmembrane region and an intracellular signaling transduction domain (167). The CAR protein engages the immune cell with the cancer cell antigen and then transform the binding signal into a signaling cascade that ultimately induce tumor killing (167). The expression of a CAR in NK cells is a promising strategy to improve anti-tumor functions by enhancing NK cells cytotoxicity and overcoming immune evasion. In contrast to CAR-T cells, CAR-NK cells transfer do no mediate GvHD, cytokine release syndrome or immune effector cell-associated neurotoxicity syndrome (169). In addition, CAR-NK cells are capable of killing malignant cells either by their intrinsic cytotoxic capacity or through the CAR-dependent mechanism (170). Predominantly CAR-NK cells have been engineered using the same constructs designed for CAR-T cells. The first generation of CARs consisted in a synthetic protein that fused an antibody-like domain to the CD3ζ chain of a TCR. Second generation CARs coupled additional co-stimulatory signaling domains, like CD28, CD134 (OX40) or CD137 (4-1BB), to improve the activation and enhance survival and expansion of the modified cells. Third-generation CARs combine two co-stimulatory domains. Fourth-generation CARs are being designed to improve the *in vivo* proliferation and persistence of immune effectors or to control potentially side effects, by for example the insertion of cytokines or suicide genes (167, 171). Few CAR constructs have been designed specifically for NK cells by incorporating DAP10 as co-stimulatory domain and NKG2D as transmembrane domain (172, 173). The therapeutic potential of CAR-NK cells in hematological malignancies is being explored in several preclinical studies and some clinical trials.

### CAR-NK Cells Pre-Clinical Development

The initial CAR-NK cells studies focused on B cell malignancies (174–176). As an example, allogenic PB NK cells were expanded and nucleofected with the anti-CD20-4-1BB-CD3ζ construct to generate anti-CD20 CAR-NK cells that improved cytotoxicity against NK cell resistant B-cell lymphoma cells *in vitro*, and also reduced tumor burden and enhanced survival in a xenograft mouse model (177). In another study, memory like NK cells were generated after stimulation of PB NK cells with IL-12, IL-15, and IL-18, and then genetically modified to express the CD19-4-1BB-CD3ζ construct. The 19-CAR-ML NK cells effectively expanded and persisted *in vivo*, and also controlled tumor growth and prolonged survival in a human xenograft model. Importantly, lymphoma patient 19-CAR-ML NK cells displayed an improved antitumor activity against autologous lymphoma cells (178). The group of Dr. Rezvani expanded and modified CB NK cells with a fourth generation CAR vector (iCasp9-CAR19-CD28-CD3ζ-IL-15) that incorporated the IL-15, to enhance CAR-NK cells proliferation and persistence, and the inducible caspase-9 (iC9) suicide gene. The iC9/CAR.19/IL-15-NK cells efficiently killed a lymphoma cell line and primary CLL leukemia cells *in vitro*, and showed long-term persistence and improved antitumor activity *in vivo* (179). Deletion of the CIS negative regulator of IL-15 signaling (CISH gene knock down) improves the metabolic fitness and antitumor efficacy of CB-NK cells (180). The CISH gene was deleted in iC9/CAR.19/IL-15-NK cells to generate CISH-/- iC9/CAR19/IL-15 CAR-NK cells. *In vivo* studies revealed that CISH-/- iC9/CAR19/IL-15 CB-NK cells persisted twice as long as control CAR19/IL-15 NK cells and significantly improved anti-tumor responses since animal treated with CISH KO iC9/CAR19/IL-15 CB-NK cells were tumor free (181).

Human iPSC-derived CAR19 NK cells (FT596) were generated by modifying the hnCD16-iNK cells (149) with the CD19-NKG2D-2B4-CD3ζ CAR construct and an IL-15 receptor fusion (IL-15RF) gene. FT596 in combination with rituximab promoted synergistic anti-tumor responses against CD19+ CD20+ B lymphoblast cells *in vitro*. Also, FT596 in combination with rituximab showed enhanced killing of tumor cells in a lymphoma mouse model, as compared to CAR19 T cells (doi.org/10.1182/blood-2019-129319). Despite the good results obtained in CD19-directed therapies in B-cell malignancies, some patients still relapse due to the loss of the CD19 antigen (182) (183). To overcome altered CD19 expression or loss of the antigen in the treatment of pre-B cell acute lymphoblastic leukemia (B-ALL), FMS-like tyrosine kinase 3 (FLT-3)-specific CAR-NK-92 cells (NK-92/4G8.28.z) were generated by transduction of NK-92 cells with an FLT3-CD28-CD3ζ vector. NK-92/4G8.28.z cells were specifically activated by FLT3+ leukemia cells *in vitro* and significantly delayed disease development in an aggressive leukemia xenograft model (184). To improve the safety of this system, the FLT3-specific CAR 4G8.28.z was co-expressed with iCasp9 an inducible caspase-9 (iCasp9) suicide gene in NK-92 cells, which resulted in the elimination of NK-92/iCasp9_2A_4G8.28.z cells upon iCasp9 activation (184).

Multiple myeloma is a heterogenous hematologic malignancy that urgently needs novel therapies as most of the patients relapse with current treatments. Preclinical data using an anti-CD138 CAR, generated after transduction of the NK‐92MI cell line (which express human IL-2) (185), with the CD138-CD3ζ vector, showed enhanced cytotoxicity on CD138+ MM cells *in vitro* and *in vivo* (186). Other study used the NK-92 cell line to express the CS1-CD28-CD3 ζ CAR construct. CS1-CAR NK cells efficiently eradicated CS1-expressing MM cells, both *in vitro* and *in vivo* (187). NKG2D is an activating receptor with broad target specificity and NKG2D-based CAR therapy could potentially target a large variety of human tumors. The efficacy of a NKG2D-CAR, generated by genetic engineering of activated and expanded NK cells (NKAE) with the NKG2D-4-1BB-CD3z-CAR construct, was analyzed in a preclinical study for MM. NKG2D-CAR-NKAE cells demonstrated to be safe and efficiently eradicated MM cells *in vitro* and displayed potent anti-tumor efficacy *in vivo* (188).

One major obstacle to targeting AML with immunotherapy is that many myeloid antigens are expressed at similar levels on normal and malignant cells. The interleukin-3 receptor alpha chain (CD123) is highly expressed on the many primary AML cells (189). The NK-92 cell line and PB NK cells were genetically modified to express the CAR construct CD123- CD28, 4-1BB-CD3ζ to generate CD123-CAR-NK. Both, CD123-CAR-NK-92 cells and CD123-CAR-NK cells, showed anti leukemic function, however CD123-CAR-NK-92 cells were more stable and had stronger cytotoxic activity against leukemia cells lines and primary AML cells (190). CD4 is an antigen expressed in M4 and M5 subtypes of AML. NK-92 cell were transduced with the CD4-CD28, 4-1BB-CD3ζ CAR vector to obtain CD4CAR NK-92 cells, which successfully eradicated CD4+ AML cells *in vitro* and demonstrated robust anti leukemic activity in a CD4+ AML xenograft mouse model (191). Other strategy to target AML cells utilized CAR NK cells expressing the activating receptor NKG2D (NKX109), generated with the CAR vector NKG2-OX40-CD3ζ, and the mbIL-15. Pre-clinical data in AML xenografts mouse models showed that NKX101 had enhanced antitumor function as compared with WT NK cells (doi.org/10.1182/blood-2020-134625)

T-cell based malignancies remain a challenge for CAR T-cell therapy since most target antigens are shared between malignant cells and the therapeutic agent, leading to CAR-T cell fratricide, therefore CAR-NK therapy is a promising alternative for T cell malignancies. CD4 is also expressed in Peripheral T-cell lymphomas (PTCLs) and CD4CAR NK-92 cells could also eliminated CD4+ T-cell leukemia and lymphoma cells *in vitro* and reduced tumor burden and prolonged survival in a T cell lymphoma xenograft model (192). CD3 is an antigen expressed in PTCLs and a small subset of T-cell acute lymphoblastic leukemia (T-ALL). CD3 CAR NK-92 cells, generated by genetic engineering of NK-92 cells using the C3-4-1BB-CD28-CD3ζ construction, specifically eliminated CD3+ lymphoma/leukemic cells, suppressed tumor growth and significantly prolonged survival in a CD3+ lymphoma xenograft model (193). Other preclinical study targeted the CD5 antigen, which is expressed in a majority of T-cell malignancies. Anti CD5-specific CAR NK cells were generated by modifying NK-92 cells with the CD5-4-1BB-CD28-CD3ζ vector. CD5 CAR-NK-92 cells displayed specific cytotoxic responses against CD5+ cell lines and CD5+ primary tumor cells *in vitro* and significantly inhibited disease progression in xenograft mouse models (194).

### CAR-NK Cell Clinical Studies

Currently, there are 15 clinical trials registered in ClinicalTrials.gov (August 2021), evaluating the safety and efficacy of CAR-NK cells in hematological malignancies, with two published studies (NCT02944162 and NCT03056339).

This fist clinical trial (NCT02944162) included only 3 patients with relapsed or refractory AML and used anti-CD33 CAR-NK cells as therapeutic agent, generated by transduction of NK-92-MI cells with the CD33-CD28-4-1BB-CD3ζ CAR construct. CD33-CAR NK-92 cell infusions were safely administrated to patients in three increasing doses; however, this study did not show durable responses (195). Dr. Rezvani’s group reported the result of a dose escalation study of HLA-mismatched CB-derived CAR-NK-CD19 cells [iC9/CAR.19/IL-15-NK cells (179)] in combination with lymphodepleting chemotherapy in 11 patients with relapsed or refractory CD19- B-cell lymphoid malignancies (NCT03056339). After infusion CAR-NK-CD19 cells expanded and persisted at low levels for at least 12 months, in the absence of neurotoxicity, CRS or GvHD. 8 out of 11 patients showed a clinical response, with 7 patients going into complete remission (169).

For the rest of the trials clinical results are currently pending. Most of the clinical are focused in B-cell malignancies and target lineage marker CD19 (9) or CD22 (2). Two studies are focused in AML/Myelodysplastic Syndromes and target CD33 or utilized NKG2D CAR-NK cells, while one study targets BCMA in MM and other CD7 in T-cell malignancies. Trials utilize NK-92 cells, allogeneic NK cells, two studies use IPSC-derived NK cells (FT596) and one CB NK cells.

## Conclusions

Since the first description, more than 50 years ago, important advances in the field of immunology has been made from studying and understanding the NK cell function. The rising of the missing self-hypothesis led us to appreciate how immune system, by mean of NK cells, is able to recognize the self and the non-self through a system that differs from other immune cells (pattern recognition receptors). This system relies on an array of receptors specific for self-MHC class I molecules, which expression is often perturbed in infected or malignant cells. These arrays of receptors are coupled to a module of inhibition that prevents unsolicited NK cell effector functions. When inhibition signals are in an Off-mode, as consequence of absence of self-MHC class I molecules, or due to a miss-match between inhibitory receptors and MHC-class I molecules, then NK cells can mobilize the cytotoxic machinery. This last scenario, is observed in the graft versus leukemia effect, and has become in an important immunotherapeutic resource for the treatment of hematological malignancies. Moreover, blocking the ligation of inhibitory receptors through monoclonal antibodies has been shown to be useful to unleash T cell responses and this option needs to be more explored in NK cells. However, a deeper understanding of the signaling mechanism, both positive and negative, that control NK cell reactivity and tolerance may allow to identify novel therapeutic targets. In addition, a better understanding of the mechanisms that regulate NK cell education may represent another important opportunity field to re-educate NK cells. In this sense, the ability of NK cells to undergo “adaptation” to new environmental clues indicates that NK cells display inherent cellular plasticity, and as consequence, this plasticity may be tuned-up to improve NK cell responsiveness. Therefore, NK cells represent a successful example of how basic research still represent our best way to generate new knowledge that can be interpretated to design and develop new therapeutic approaches against hematological malignancies and perhaps other cancers.

## Authors Contributions

VR-M write the manuscript; JG write the manuscript; JA write the manuscript; MC-M conceive and write review. All authors contributed to the article and approved the submitted version.

## Funding

This study was supported by grant 241854 (to MC-M) from CONACYT; grant 377883 (to MC-M) from CONACYT; Grants CB-2015-258042-M and FONCICYT/37/2018) (to JA) from CONACYT.

## Conflict of Interest

The authors declare that the research was conducted in the absence of any commercial or financial relationships that could be construed as a potential conflict of interest.

## Publisher’s Note

All claims expressed in this article are solely those of the authors and do not necessarily represent those of their affiliated organizations, or those of the publisher, the editors and the reviewers. Any product that may be evaluated in this article, or claim that may be made by its manufacturer, is not guaranteed or endorsed by the publisher.
